# Genome-wide characterization and expression analysis of the heat shock transcription factor family in pumpkin (*Cucurbita moschata*)

**DOI:** 10.1186/s12870-020-02683-y

**Published:** 2020-10-14

**Authors:** Changwei Shen, Jingping Yuan

**Affiliations:** 1 School of Resources and Environmental Sciences, Henan Institute of Science and Technology, Xinxiang, 453003 China; 2 School of Horticulture and Landscape Architecture, Henan Institute of Science and Technology, Xinxiang, 453003 Henan China; 3 Henan Province Engineering Research Center of Horticultural Plant Resource Utilization and Germplasm Enhancement, Xinxiang, 453003 China

**Keywords:** *Cucurbita moschata*, Heat shock transcription factor, Gene duplication, Conserved domain, *Cis*-acting elements, Expression pattern

## Abstract

**Background:**

Crop quality and yield are affected by abiotic and biotic stresses, and heat shock transcription factors (*Hsfs*) are considered to play important roles in regulating plant tolerance under various stresses. To investigate the response of *Cucurbita moschata* to abiotic stress, we analyzed the genome of *C. moschata*.

**Results:**

In this research, a total of 36 *C. moschata Hsf* (*CmHsf*) members were identified and classified into three subfamilies (I, II, and III) according to their amino acid sequence identity. The *Hsfs* of the same subfamily usually exhibit a similar gene structure (intron-exon distribution) and conserved domains (DNA-binding and other functional domains). Chromosome localization analysis showed that the 36 *CmHsfs* were unevenly distributed on 18 of the 21 chromosomes (except for Cm_Chr00, Cm_Chr08 and Cm_Chr20), among which 18 genes formed 9 duplicated gene pairs that have undergone segmental duplication events. The Ka/Ks ratio showed that the duplicated *CmHsfs* have mainly experienced strong purifying selection. High-level synteny was observed between *C. moschata* and other *Cucurbitaceae* species*.*

**Conclusions:**

The expression profile of *CmHsfs* in the roots, stems, cotyledons and true leaves revealed that the *CmHsfs* exhibit tissue specificity. The analysis of *cis*-acting elements and quantitative real-time polymerase chain reaction (qRT-PCR) revealed that some key *CmHsfs* were activated by cold stress, heat stress, hormones and salicylic acid. This study lays the foundation for revealing the role of *CmHsfs* in resistance to various stresses, which is of great significance for the selection of stress-tolerant *C. moschata*.

## Background

Plants are constantly subjected to all kinds of adverse environmental pressures during growth and development stages, thus, they have developed special mechanisms to cope with adverse conditions [1, 2]. Transcription factors usually play an important role in the regulation of stress responses [3]. Heat shock transcription factors (*Hsfs*) are the most important transcription regulators [4]. They are the terminal components of signal transduction chains and can mediate the activation of genes that respond to various abiotic pressures (drought stress, heat stress and a large number of chemical stress factors) [4].

The first *Hsf* gene was cloned from yeast [5, 6], followed by some mammals [7–10]. The first plant *Hsf* gene was cloned from tomato [11]. With the sequencing of the *Oryza sativa* and *Arabidopsis thaliana* genomes, *Hsf* genes have also been identified in *O. sativa* and *A. thaliana* [12, 13]. Subsequently, researchers identified 31, 25, 21, 26, 35, 29, 27, 19 and 35 *Hsf* genes in the *Populus trichocarpa* [14], *Zea mays* [15], *Cucumis sativa* [16], *Glycine max* [17], *Brassica rapa ssp. pekinensis* [18], *Pyrus bretschneideri* [19], *Solanum tuberosum* [20], *Vitis vinifera* [21] and *Brassica oleracea* [22] genomes, respectively.

A typical Hsf usually contains four conserved domains: a DNA-binding domain (DBD) at the N-terminus, a hydrophobic oligomerization domain (HR-A/B or OD), a nuclear localization signal (NLS), and a nuclear export signal (NES) [23]. The DBD is the most conserved domain structure in Hsfs and is mainly responsible for binding to the heat shock elements (HSEs) of the target gene promoter, while the HR-A/B domain is a hydrophobic heptad repeat forming a spiral coil structure, which is a prerequisite for transcription [23]. The NLS is rich in Arg (R) and Lys (K) residues, while the NES is rich in Leu (L). NLS is recognized by the corresponding NES, which interacts with nucleoporins to help protein containing nuclear localization signal reach the nucleus through the nuclear pore [24–26]. There is a flexible link between the DBD and the HR-A/B domain. Based on the structural characteristics of the conserved DBD and HR-A/B domain, the *Hsfs* have been divided into three groups (A, B and C). The main differences between the three groups are as follows: group B proteins exhibit 7 amino acid residues in their HR-A/B domain, while group A has 28 amino acid residues in the relevant domain and group C had 14 amino acid residues in the same domain. In addition, the transcription activation domain (AHA) at the C-terminus is characteristic of group A, which guarantees the normal transcription of the *Hsfs* by binding to some basic transcription protein complexes. However, the *Hsfs* of group B and group C cannot maintain their activation activity due to the lack of an AHA motif [26, 27]. The repression domain (RD) is a peptide containing conserved amino acids (LFGV) at the C-terminus and mainly exists in group B [28].

Hsfs can specifically regulate the transcription of heat shock protein (*Hsp*) genes by specifically binding to the HSE in the promoter of an *Hsp* gene, and the *Hsp*, in turn, protect cells from stress and participate in protein folding [29, 30]. Some studies have confirmed that *Hsfs* are involved in the heat stress response. For example, the silencing of *HsfA1a* in tomato reduces the synthesis of heat stress-induced chaperone and HsfA1a proteins, thereby increasing the sensitivity of *HsfA1a*-silenced tomato plants to heat stress [31]. At 37 °C, *A. thaliana HsfA2*-mutant plants are more sensitive to heat stress than wild-type plants, which can be reversed by introducing the *HsfA2* gene [32]. The *OsHsfA4d-*mutant shows a phenotype of necrotic damage under high-temperature stress [13]. The expression of *OsHsfA2e* enhances high temperature and salt tolerance in *A. thaliana* [33]. In addition to heat stress, *Hsfs* are involved in plant growth and other biotic and abiotic stress responses. It is found that *HsfA9* is involved in embryo development and seed maturation in *A. thaliana* and *Helianthus annuus* [34]. Four *Hsf* genes (*HsfA1e*, *HsfA3*, *HsfA4a*, *HsfB2a* and *HsfC1*) in *A. thaliana* are strongly induced by salt, cold and osmotic stress [35–37]. The *HsfA2* in *A. thaliana* is involved in the response to oxidative stress [38]. The *HsfA4a* in *A. thaliana* can be used as an H_2_O_2_ sensor [35, 39]. The *OsHsfA4a* in *O. sativa* is associated with cadmium tolerance [40]. To date, there have been no reports of the cloning and functional analysis of *Cucurbita moschata Hsfs*.

*C. moschata* is rich in a variety of amino acids, vitamins, polysaccharides, pectin, and minerals and contains trigonelline, carotenoids and other biologically active substances and nutrients [41]. According to the Food and Agriculture Organization of the United Nations (http://www.fao.org/home/en/), pumpkin ranks the ninth in the output value of different vegetable crops in the world, with an annual sales value of 4 billion US dollars. China and India are the two main pumpkin producing countries in the world. China’s cultivation area ranks second in the world, and its total output ranks first in the world [42]. During growth and development, unfavorable stress often causes great harm to the growth of pumpkin, resulting in a decline in pumpkin yield and quality [41]. Therefore, research on pumpkin resistance-related genes is increasingly important for pumpkin breeding and production. Because the *C. moschata* (*Rifu*) genome has been published [43], the Hsf family in *C. moschata* can now be subjected to systematic and comprehensive analysis. In this study, we provide information about the gene structural characteristics, gene duplications, chromosomal locations, evolutionary divergence and phylogenetic relationships of 36 *C. moschata Hsf* genes. Furthermore, we analyze the digital expression profiles of 36 *CmHsfs* in response to numerous stresses. This study emphasizes the function of the *Hsfs* in various stress conditions and improves our understanding of the effects of polyploidization events on the evolution of the Hsf family.

## Results

### Identification of *Hsf* genes in *C. moschata* and their physical and chemical characteristics

A total of 36 *CmHsf* genes were identified after the removal of false positives and the same genes (Table 1), and they were designated *CmHsf1* to *CmHsf36* according to the starting positions of these genes on the chromosomes (from Cmo_Chr00 to Cmo_Chr20, from top to bottom). The physicochemical parameters of each *CmHsf* were generated, and the predicted open reading frames (ORFs) ranged from 543 bp (*CmHsf32*) to 4380 bp (*CmHsf13*), with predicted proteins of 179–1458 amino acids. The physical and chemical parameters of these genes are similar to those seen in *A. thaliana* and *O. sativa* [44]. Furthermore, the molecular weights (MW) of these *CmHsfs* ranged from 20.5642 to 161.5554 kDa (*kDa*) (Table 1). Although the deduced heat shock transcription factors presented diversity in terms of the parameters mentioned above, most of the *CmHsfs* exhibited low isoelectric points (*pI*) (average 6.3) (Table 1). Subcellular localization prediction indicated that only 2 heat shock transcription factors (CmHsf12 and CmHsf17) were predicted to be localized to the cell membrane, cytoplasm and nucleus, while the remaining CmHsfs were predicted to be localized to the nucleus.

**Table 1:** Physical and chemical characteristics of the 36 *Hsf* genes identified in *Cucurbita moschata*

Gene ID	Gene name	Cmo_Chr ^a^	Start^b^	End^c^	ORF length (bp)	AA^d^	*pI*^e^	Mw^f^ (Da)	Loc^g^
CmoCh01G018910.1	*CmHsf01*	01	13,630,401	13,636,203	1701	565	7.32	63,908.05	Nucleus.
CmoCh02G000520.1	*CmHsf02*	02	279,098	280,430	945	313	6.23	35,866.58	Nucleus.
CmoCh02G015130.1	*CmHsf03*	02	8,829,467	8,831,346	1017	337	4.79	37,042.37	Nucleus.
CmoCh03G000560.1	*CmHsf04*	03	917,233	919,195	723	239	9.35	27,525.06	Nucleus.
CmoCh03G009950.1	*CmHsf05*	03	7,477,236	7,479,691	900	298	5.6	33,388.41	Nucleus.
CmoCh03G012560.1	*CmHsf06*	03	9,632,303	9,635,635	1392	462	7.55	52,807.71	Nucleus.
CmoCh04G000850.1	*CmHsf07*	04	461,682	465,859	1218	404	4.88	46,844.9	Nucleus.
CmoCh04G011130.1	*CmHsf08*	04	5,675,420	5,678,524	1134	376	4.95	43,681.38	Nucleus.
CmoCh05G000960.1	*CmHsf09*	05	393,383	395,093	1110	368	4.93	41,839.89	Nucleus.
CmoCh05G001750.1	*CmHsf10*	05	759,147	761,562	1362	452	7.64	50,399.85	Nucleus.
CmoCh05G013450.1	*CmHsf11*	05	10,456,658	10,458,207	993	329	6.12	37,483.5	Nucleus.
CmoCh05G014000.1	*CmHsf12*	05	10,787,694	10,799,787	3714	1236	6.8	139,325.5	Cell membrane. Cytoplasm. Nucleus.
CmoCh06G004420.1	*CmHsf13*	06	2,118,798	2,130,108	4380	1458	5.55	161,555.42	Nucleus.
CmoCh06G006450.1	*CmHsf14*	06	3,242,367	3,246,508	1566	520	5.12	57,039.44	Nucleus.
CmoCh06G009230.1	*CmHsf15*	06	6,678,383	6,679,150	687	227	8.85	26,518.21	Nucleus.
CmoCh06G012330.1	*CmHsf16*	06	9,329,887	9,333,367	1416	470	6.48	52,376.05	Nucleus.
CmoCh06G013840.1	*CmHsf17*	06	10,166,157	10,173,534	1650	548	5.35	64,159.97	Cell membrane. Cytoplasm. Nucleus.
CmoCh07G001570.1	*CmHsf18*	07	853,089	854,975	1227	407	5.45	46,937.23	Nucleus.
CmoCh07G002420.1	*CmHsf19*	07	1,191,784	1,192,862	579	191	8.38	22,514.5	Nucleus.
CmoCh07G007220.1	*CmHsf20*	07	3,258,238	3,259,253	873	289	6.36	32,815.78	Nucleus.
CmoCh09G002330.1	*CmHsf21*	09	1,070,417	1,071,523	993	329	8.56	37,629.07	Nucleus.
CmoCh10G006520.1	*CmHsf22*	10	2,987,379	2,988,593	855	283	6.07	32,230.65	Nucleus.
CmoCh10G009220.1	*CmHsf23*	10	4,574,443	4,576,160	750	248	8.75	28,527.16	Nucleus.
CmoCh13G006110.1	*CmHsf24*	11	6,682,088	6,683,686	1239	411	5.21	46,658.15	Nucleus.
CmoCh11G009050.1	*CmHsf25*	11	4,658,284	4,659,725	708	234	7.96	27,047.37	Nucleus.
CmoCh12G005810.1	*CmHsf26*	12	3,595,429	3,596,964	1074	356	4.88	40,558.39	Nucleus.
CmoCh11G006110.1	*CmHsf27*	13	2,932,186	2,933,416	879	291	5.61	33,180.33	Nucleus.
CmoCh14G002670.1	*CmHsf28*	14	1,203,588	1,210,628	2073	689	5.78	76,886.63	Nucleus.
CmoCh14G017830.1	*CmHsf29*	14	13,739,183	13,747,848	2604	866	5.34	97,900.69	Nucleus.
CmoCh14G019680.1	*CmHsf30*	14	14,515,610	14,518,497	1350	448	6.53	50,396.1	Nucleus.
CmoCh15G012680.1	*CmHsf31*	15	8,690,633	8,692,333	1059	351	4.64	39,145.25	Nucleus.
CmoCh16G001410.1	*CmHsf32*	16	644,769	646,828	543	179	8.42	20,564.2	Nucleus.
CmoCh16G012250.1	*CmHsf33*	16	8,775,979	8,782,264	1572	522	4.9	57,215.79	Nucleus.
CmoCh17G011810.1	*CmHsf34*	17	9,496,232	9,498,290	1140	378	4.9	43,615.67	Nucleus.
CmoCh18G012590.1	*CmHsf35*	18	12,324,683	12,328,013	1059	351	5.77	39,472.8	Nucleus.
CmoCh19G000190.1	*CmHsf36*	19	124,488	127,854	1164	386	5.78	44,382.64	Nucleus.

Note: Information on including their chromosomal distribution, their start and the end positions on the chromosomes, nucleic acid sequence and amino acid sequence were extracted from *Cucurbit* genomics database, and all the data in the table is predicted or theoretical

^a^ Cmo_Chr,The name of the CmHsf chromosome corresponding to the gene

^b^ Start, Predicted starting position of mRNA

^c^ End, Predicted termination position of mRNA

^d^ AA, Amino acid number in CmHsf protein sequences

^e^ *pI*, Theoretical Isoelectric point

^f^ MW, Molecular weight (Mw) predicted by ExPASy (http://web.expasy.org/tools/)

^g^ Loc, Subcellular location of the CmHsf proteins predicted by Plant-mPLoc

### Classification and conserved domain analysis of 36 CmHsfs

**Fig. 1:**
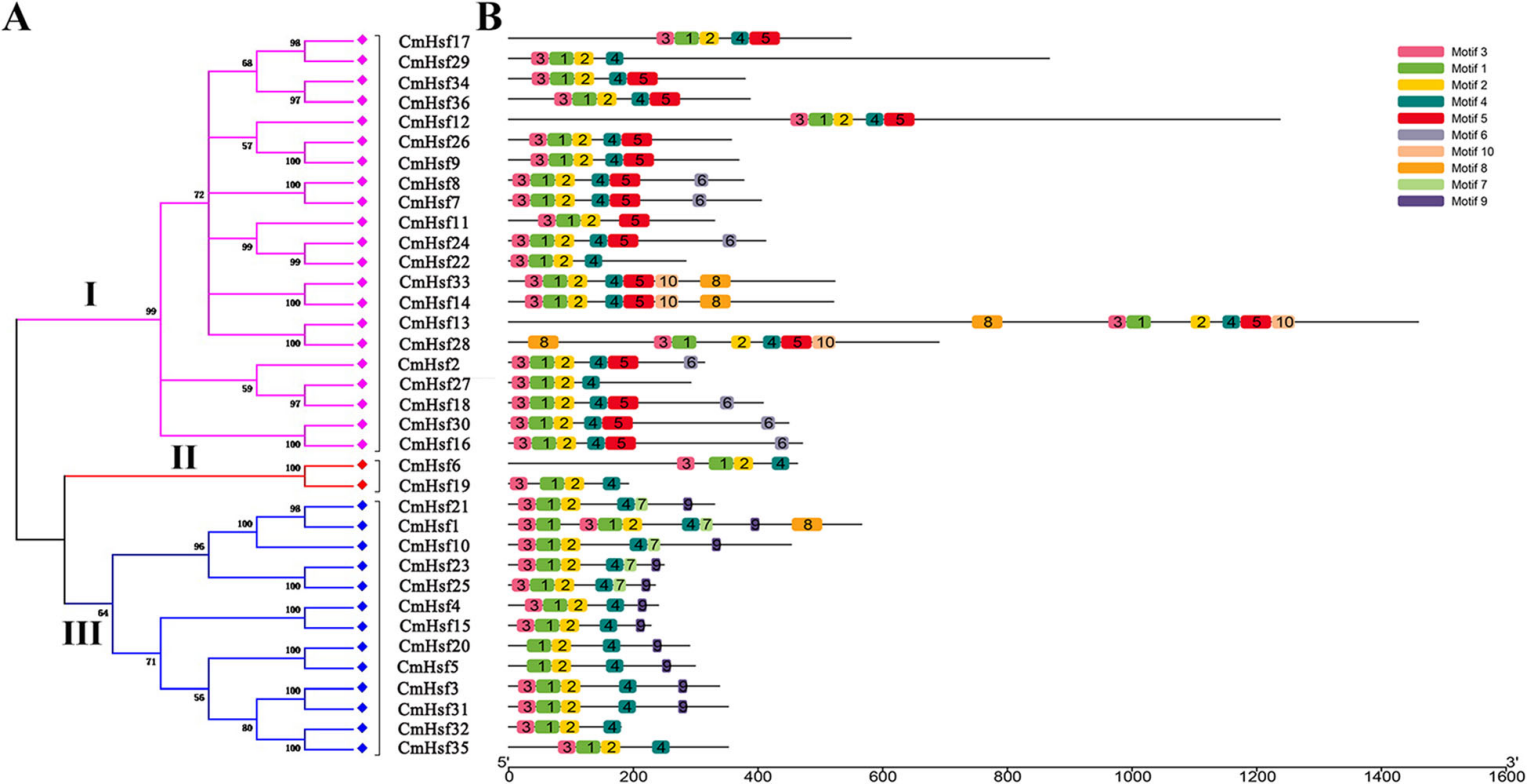
Classification and conserved motifs of 36 *CmHsfs*. **a.** The unrooted phylogenetic tree of 36 *CmHsfs* was constructed using the Neighbor-joining (NJ) method with 1000 bootstrap replicates, and a 60% cut-off value was used for the condensed tree. Three different subfamilies (I-III) were highlighted with different colored branch lines. **b.** Schematic representation of conserved motifs in 36 *CmHsfs*. Each motif was represented by a numbered colored box on the right. The same number in different proteins referred to the same motif. Motif 1, motif 2 and motif 3 together formed the DBD, and motif 4 formed the HR-A/B domain. The function of other motifs was unknown

To identify the phylogenetic relationships of the 36 CmHsfs, an unrooted phylogenetic tree was produced. These CmHsfs can be divided into three subfamilies (subfamily I, subfamily II and subfamily III; Fig. 1a) according to the amino acid sequence identity. Subfamily I (containing 21 members) was the largest group, and subfamily III included 13 members, while subfamily II presented the fewest members (2 members) (Fig. 1a). Furthermore, based on the structural characteristics of the conserved DBDs and HR-A/B domains, we can divide the 36 *CmHsfs* into three groups (A, B, and C) (Table 2). All CmHsfs contained a DBD and an HR-A/B domain (Table 2), and the DBD was composed of approximately 100 conserved amino acids (Additional file 2: Fig. S1). In addition, except for CmHsf27 and CmHsf32, all of the CmHsfs contained an NLS. The CmHsfs in group A contained an AHA domain, while the CmHsfs in groups B and C did not contain an AHA domain, and only the proteins in Group B contained an RD (Table 2). To further reveal conserved domains, all CmHsfs were submitted to MEME, and 10 different motifs were identified (Fig. 1b; Additional file 2: Fig. S2). Overall, the CmHsfs exhibited 4–9 motifs, and motifs 1, 2 and 4 were present in all CmHsf proteins. Motif 3 was present in all proteins except for CmHsf20 and CmHsf5. In addition, we found that motif 5 existed only in subfamily I, while motif 9 appeared only in subfamily III (Fig. 1b). The CmHsfs from the same clade usually present conserved domains or similar motif compositions, suggesting functional similarities among these proteins.

**Table 2:** Functional domain analysis of the 36 Hsf proteins identified in *Cucurbita moschata*

Subfamily Name	Gene ID	Gene Name	Group	DBD	HR-A/B	NLS	NES	AHA	RD
Subfamily I	CmoCh06G013840.1	CmHsf17	A	243–336	359–407	(428)QKDKHKELEEAINRKRRRHI	nd	DDGFWENLL	nd
	CmoCh14G017830.1	CmHsf29	A	42–135	162–205	(225)GFRKVDPDKWEFAHESFLRGQRHLLKLIRR	IEELCLSL	SDFWQKLIEL	nd
	CmoCh17G011810.1	CmHsf34	A	43–136	163–206	(243)ITRKRRRPIQ	TELEALALEMQGL	EGFWEELFSE	nd
	CmoCh19G000190.1	CmHsf36	A	79–172	198–242	(278)ATKKRRWPID	LEALAMEM	EGFWEEFFSE	nd
	CmoCh05G014000.1	CmHsf12	C	458–551	573–599	(1191)QRRPPVGPEDPKRSASGRHTGYVKNYD	nd	nd	nd
	CmoCh12G005810.1	CmHsf26	C	39–132	153–205	(236)RKRRLTASPSLENLQDETILAAVKQEQLE	nd	nd	nd
	CmoCh05G000960.1	CmHsf9	C	41–134	155–208	(236)EIGRKRRLTSS	nd	nd	nd
	CmoCh04G011130.1	CmHsf8	C	12–105	137–184	(323)IDHEKRSVDNEDDELDMETIDTRTHEEKSQD	nd	nd	nd
	CmoCh04G000850.1	CmHsf7	C	12–105	137–184	(369)RLDESYIEKSNTVNLMELMASDQEILYETPAKMQG	nd	nd	nd
	CmoCh05G013450.1	CmHsf11	A	53–146	162–195	(221)RRVRRRVTMRPPPSPVKFVKA	VKREDDGELALEISKLKQEQI	SNFWDDLLVQ	nd
	CmoCh13G006110.1	CmHsf24	A	11–104	124–172	(187)RFLHKPGLRLDLLPQLETSDRKRRLP	LKRDKEQLLLELRKHEQ	DVFWQQFLTE	nd
	CmoCh10G006520.1	CmHsf22	A	9–102	122–155	(189)PDKKRRFMTS	nd	EGFWEELFSE	nd
	CmoCh16G012250.1	CmHsf33	A	32–125	154–205	(236)EANKKRRLKQD	MKVLLDEKLCLDNH	SNFWDDLLVQ	nd
	CmoCh06G006450.1	CmHsf14	A	32–125	154–205	(236)EANKKRRLKQD	LQDFELLIKQM	SNFWNDLLVH	nd
	CmoCh06G004420.1	CmHsf13	A	968–1124	1144–1195	(1225)PRMKRKFVKQ	LQLALALRL	LSPFWDLGSL	nd
	CmoCh14G002670.1	CmHsf28	A	239–387	407–438	(474)FLLKRKKEPKDIDSERIKRKFVK	nd	DVFWEQFLTE	nd
	CmoCh02G000520.1	CmHsf2	A	11–104	117–155	(173)RMGNQQKQLIAIMAAELQKDQSRKRRK	LSELERQELELKI	DVFWEQFLTE	nd
	CmoCh11G006110.1	CmHsf27	A	11–104	117–155	nd	LEEELEGM	DVFWEQFLTE	nd
	CmoCh07G001570.1	CmHsf18	A	11–104	123–179	(205)HERKRRLATV	LQLQMQL	DVFWEQFLTE	nd
	CmoCh14G019680.1	CmHsf30	A	9–102	119–174	(201)FNKKRRLPS	LQLQELTM	DVFWEQFLTE	nd
	CmoCh06G012330.1	CmHsf16	A	14–107	124–179	(206)FNKKRRLPS	IQLQDLTV	DVFWEQFLTE	nd
Subfamily II	CmoCh03G012560.1	CmHsf6	C	276–391	320–352	(432)RRQKLELQAQIAQFKALHIRLLDCVGRRIEK	nd	nd	nd
	CmoCh07G002420.1	CmHsf19	C	8–120	147–178	(182)KTRNPAPFLSKTY	nd	nd	nd
Subfamily III	CmoCh09G002330.1	CmHsf21	B	21–114	117–155	(287)IHSKKRLHPEYASNNIGKENNNKARFV	LEKDDLGLNL	nd	KLFGVAI
	CmoCh01G018910.1	CmHsf1	B	120–213/21–114	117–156	(374)GSSKSFVTIVEEPKTKLFGVSLQSKKRVHPE	VLEKDDLGLNL	nd	KLFGVSL
	CmoCh05G001750.1	CmHsf10	B	21–114	117–157	(337)KKRQHPDTTNYVSTSSNVSDTNKNSRGS	LLLLFKPRL	nd	KLFGVPL
	CmoCh10G009220.1	CmHsf23	B	21–114	117–158	(273)RGKKRMHHE	KQLLLAI	nd	KLFGVPI
	CmoCh11G009050.1	CmHsf25	B	11–104	124–172	(221)RGKKRGASDEE	nd	nd	KLFGVPI
	CmoCh03G000560.1	CmHsf4	B	32–125	150–187	(197)GSRKEDEDERPKLFGVRLEVEGERRRKTKR	nd	nd	KLFGVRL
	CmoCh06G009230.1	CmHsf15	B	19–112	144–180	(196)EMMVMKPNLKLFGVKLEVGEEDEMVRQSKR	LKLFGVKLEV	nd	KLFGVKL
	CmCh07G007220.1	CmHsf20	B	6–99	144–183	(244)EKNNDKNKTKREEEEKVEVCGNEPEAKVMKT	nd	nd	KLFGVWL
	CmoCh03G009950.1	CmHsf5	B	6–99	150–188	(258)EKKKMKRVREEKIGCSNAPHAKAMK	nd	nd	KLFGVWL
	CmoCh02G015130.1	CmHsf3	B	21–114	177–207	(254)FLTKTYQLVDDPDVDDLISWNEDGSTFIVW	nd	nd	KLFGVSI
	CmoCh15G012680.1	CmHsf31	B	21–114	176–206	(279)IGVKRRREEE	nd	nd	KLFGVSI
	CmoCh16G001410.1	CmHsf32	B	19–112	134–173	nd	LASAKSLDL	nd	KLFGVWL
	CmoCh18G012590.1	CmHsf35	B	85–178	226–260	(269)ENQLKSSCKVRESVLASAKSLDLFPLKRRSEE	LASAKSLDL	nd	KLFGVS

Note: The amino acid sequences of Hsf in the table came from *Cucurbit* genomics database. *DBD* DNA-binding domain, *HR-A/B* Oligomerization domain, *NLS* Nuclear localization signal, *NES* Nuclear export signal, *AHA* Transcriptional activation domain, *RD* Repressor domain; *nd* No motifs detectable by sequence similarity search. For the NLS column, the numbers in parenthesis are the start site of the functional domain

### Exon-intron analysis of 36 *Hsfs* in *C. moschata*

**Fig. 2:**
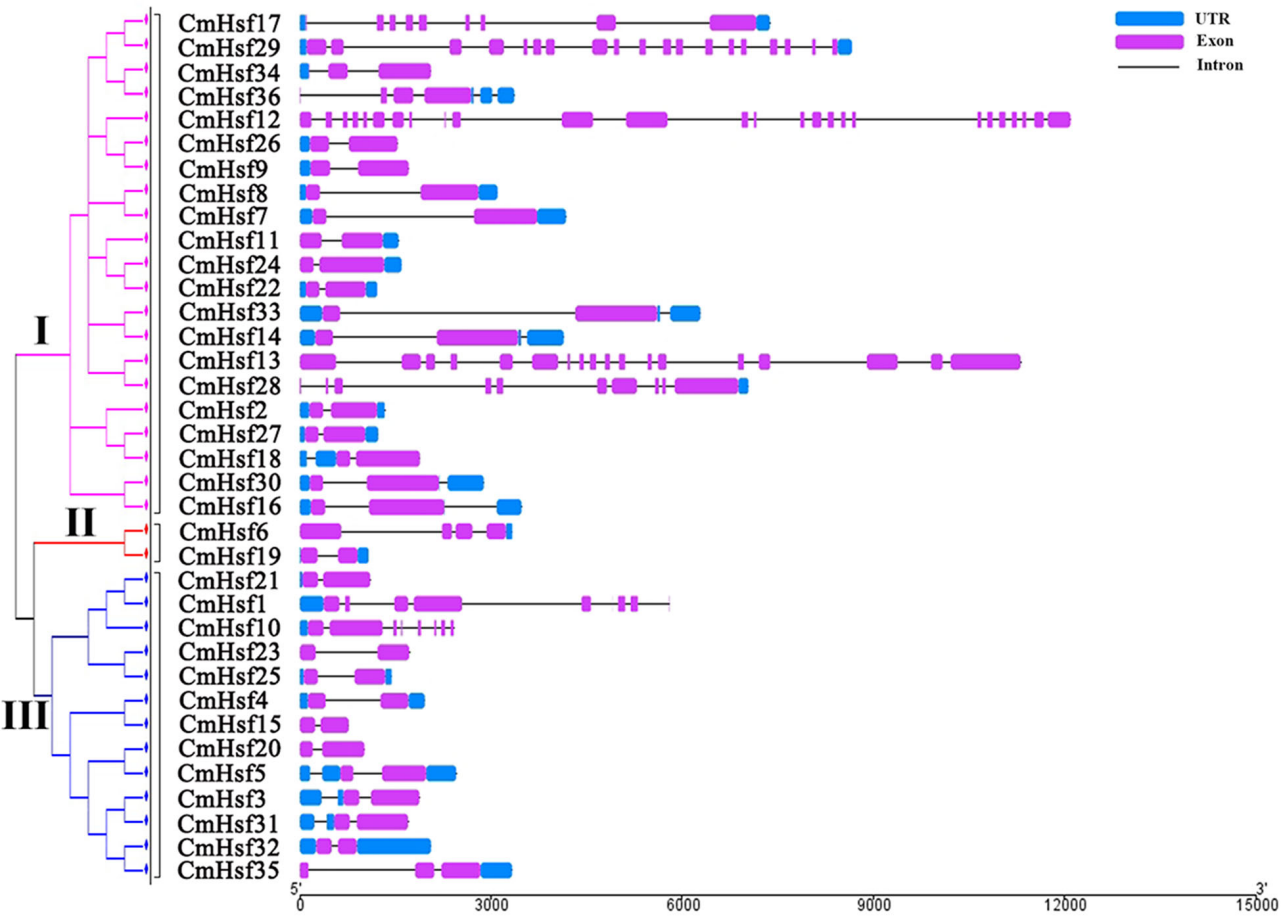
Exon-intron organization of 36 *CmHsfs* constructed by GSDS (Gene structure display server). The exons and introns were represented by pink boxes and grey lines, respectively. Untranslated regions (UTRs) were indicated by blue boxes. The sizes of the exons and introns can be estimated using the scale at the bottom

An exon-intron organization map of the 36 *CmHsf g*enes was also produced (Fig. 2). Different numbers of exons (from 2 to 26) were found in the 36 *CmHsf* genes, suggesting that *CmHsfs* are quite diverse. In subfamily III, except for *CmHsf1*, *CmHsf10 and CmHsf35*, which contained 9, 8 and 3 exons, respectively, the other *CmHsf* genes all contained 2 exons. *CmHsf* genes on the same branch usually presented similar intron-exon distributions, such as *CmHsf26*_*CmHsf9*. Some genes in the same family exhibited significantly different intron-exon distributions. For example, *CmHsf12* contained 26 exons, which was different from the other *CmHsfs*, indicating that *CmHsf12* may have a special function.

### Chromosomal distribution and gene duplication of *Hsf* genes in *C. moschata*

**Fig. 3:**
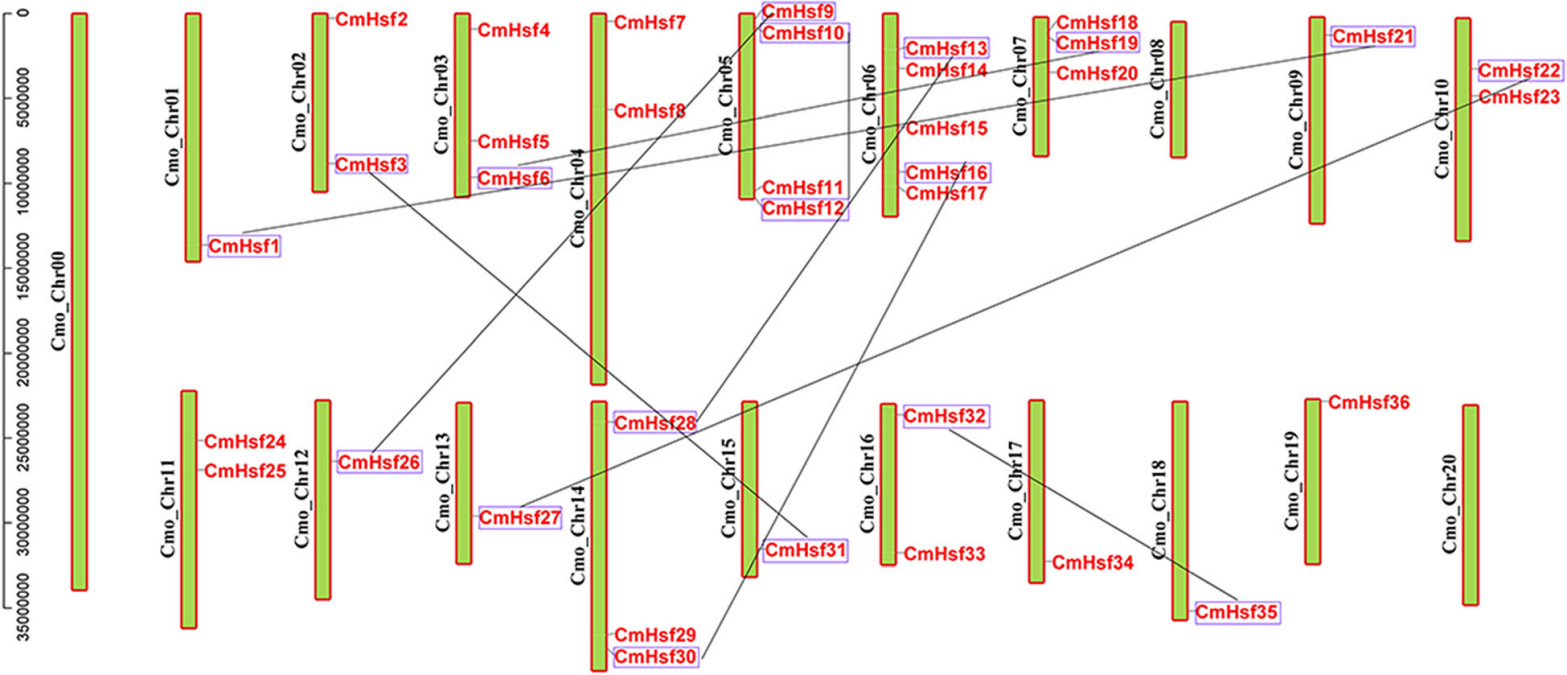
Chromosomal distribution and duplication events of *Hsf* genes in *C. moschata*. The chromosomal locations of the *CmHsf* genes were mapped with visualization tools. The duplicated *CmHsf* genes were shown in blue boxes and black lines

Chromosomal distribution analysis in the genome revealed that the 36 *CmHsf* genes were unevenly distributed on 19 of the 21 chromosomes (Fig. 3). The chromosome Cm_Chr06 exhibited the most *CmHsf* genes, with 5 genes, followed by chromosome Cm_Chr05, with 4 genes. A total of 3 genes were present on each of chromosomes Cm_Chr03, Cm_Chr07 and Cm_Chr14, and 2 genes were present on each of chromosomes Cm_Chr02, Cm_Chr04, Cm_Chr10, Cm_Chr11 and Cm_Chr16, while no genes were distributed on chromosomes Cm_Chr00, Cm_Chr08 and Cm_Chr20.

Two genes, whose putative amino acid identity is > 85% and gene alignment coverage is > 0.75, were defined here as a recently duplicated gene pair [45, 46]. A total of 18 duplicated genes were identified and divided into nine groups, each of which contained two duplicated genes. Eight duplicated gene pairs were distributed on different chromosomes (Fig. 3), which demonstrated that segmental duplication events were involved in the expansion of the *CmHsf* genes. *CmHsf10* and *CmHsf12* were separated by a region of more than 100 kb, indicating that all duplicated gene pairs had undergone segmental duplication events. The *Ka* and *Ks* ratios were less than 1.0, which suggested that the pairs had evolved mainly under functional constraints with negative or purifying selection (Table 3). We also calculated evolutionary times and divergence times of the duplicated *C. moschata Hsf* gene pairs ranging from 10.17 to 65.74 million years ago (Mya), averaging 21.11 Mya (Table 3).

**Table 3:** KaKs calculation and estimated divergence time for the duplicated *CmHsf* gene pairs

Duplicated *CmHsf* gene pairs	Identity (%)	E-value	Gene alignment coverage	*Ka*	*Ks*	*Ka*/*Ks*	Divergence time (MYA)
CmHsf12-CmHsf10	95.12	1.00E-12	0.975	0.832	1.972	0.422	65.742
CmHsf26-CmHsf9	95.12	1.00E-12	0.910	0.126	0.462	0.273	15.416
CmHsf22-CmHsf27	85.09	0	0.893	0.145	0.673	0.215	22.432
CmHsf13-CmHsf28	85.09	0	0.946	0.232	0.436	0.531	14.535
CmHsf30-CmHsf16	86.13	0	0.931	0.074	0.305	0.242	10.168
CmHsf6-CmHsf19	86.13	0	0.863	0.080	0.336	0.238	11.204
CmHsf21-CmHsf1	87.83	0	0.812	0.066	0.542	0.121	18.083
CmHsf3-CmHsf31	87.83	0	0.814	0.106	0.431	0.246	14.354
CmHsf32-CmHsf35	87.97	0	0.981	0.181	0.542	0.335	18.053

Note: We used the KaKs calculator to calculate the Ka/Ks. *Ks*, synonymous substitutions; *Ka*, nonsynonymous substitutions

### Phylogenetic relationship of Hsfs in *C. moschata*, *C. sativa* and *A. thaliana*

**Fig. 4:**
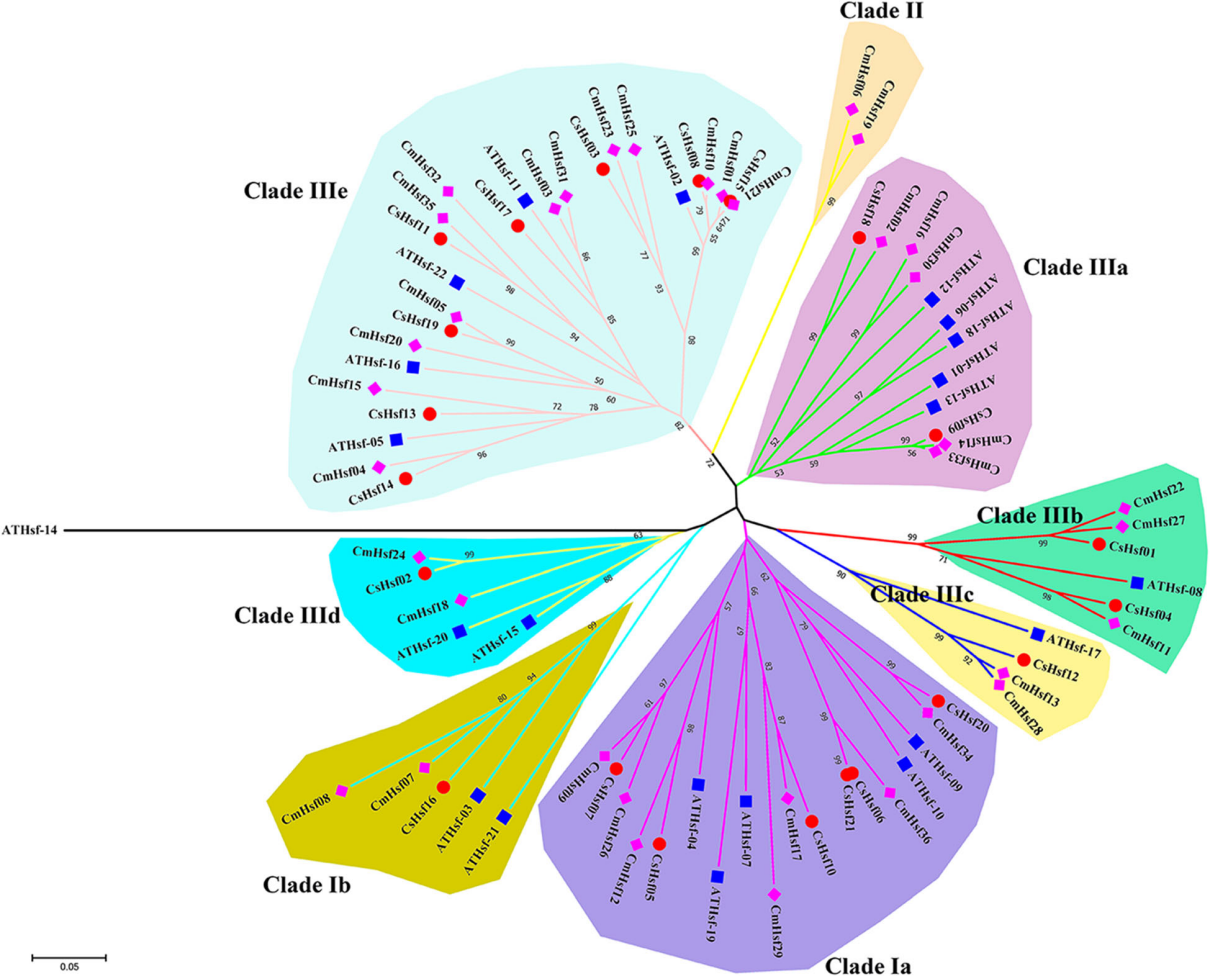
Phylogenetic trees of the *Hsf* gene family in *C. moschata*, *C. sativa* and *A. thaliana*. The 9 clades (Clade Ia-b, Clade II and Clade IIIa-e) were displayed with different background colors. The phylogenetic tree was constructed with MEGA 5.0 software using the Neighbor-joining (NJ) method with 1000 bootstrap replicates. Cm, *C. moschata*; Cs, *C. sativa*; At, *A. thaliana*

To better evaluate the molecular evolution and phylogenetic relationship of plant Hsf, a phylogenetic tree of 79 Hsf proteins in *C. moschata*, *C. sativa* and *A. thaliana* was established. Based on the previous classification of *C. moschata* Hsf proteins (Fig. 1a), they were divided into 9 clades (Clade Ia-b, Clade II and Clade IIIa-e) (Fig. 4). Subfamily I was divided into Clade Ia and Clade Ib, and subfamily III was divided into Clade IIIa-e. This classification was consistent with the phylogenetic classification of AtHsf proteins [44]. In general, genes from subfamily I (Clade Ia and Clade Ib) (including 51 Hsfs) constituted the largest branch and accounted for 65% of the total Hsfs. Subfamily II contained 2 proteins. The remaining Hsfs belong to subfamily III and contain a total of 26 Hsf proteins. From the perspective of phylogenetic branch, the homology of Hsfs between *C. moschata* and *C. sativa* was higher than that between *C. moschata* and *A. thaliana*, which was consistent with the evolutionary rules of the three species.

### Synteny analysis of *Hsf* genes in *C. moschata*

**Fig. 5:**
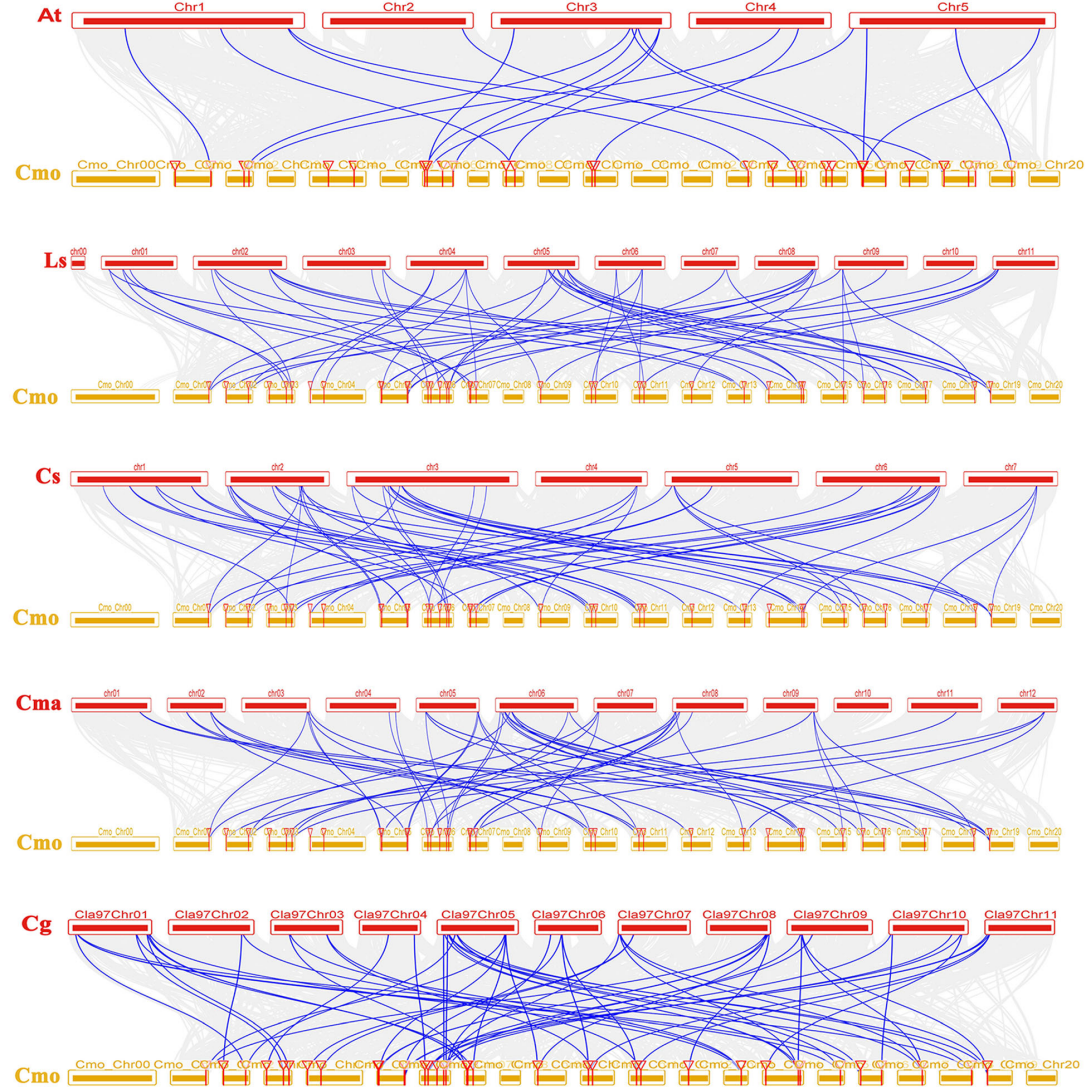
Synteny analysis of the *Hsf* genes between *C. moschata* and five other species*.* The synteny relationship maps were constructed using the Advanced Circos program in TBtools. *At*, *A. thaliana*; *Ls*, *L. siceraria*; *Cs*, *C. sativus*; *Cma*, *C. maxima*; *Cg*, *C. lanatus*; *Cmo*, *C. moschata*. The gray lines in the background indicated the collinear blocks in the genome of *C. moschata* and other plants, while blue lines in the background highlighted syntenic *Hsf* gene pairs. All the data for the various species was extracted from *Cucurbit* genomics database

According to the synteny analysis of *Hsfs* in *C. moschata* and 5 other species (*A. thaliana*; *Lagenaria siceraria*; *Cucumis sativus*; *Cucurbita maxima*; *Citrullus lanatus*), we found that *C. lanatus* exhibited the most *Hsf* homologous genes (56), followed by *L. siceraria* (52), *C. maxima* (51) and *C. sativus* (51). *A. thaliana* presented the fewest (18) homologous genes (Fig. 5). Furthermore, the syntenic genes of the *CmHsfs* could be found on all chromosomes of *A. thaliana*, *L. siceraria*, *C. sativus*, *C. maxima*, and *C. lanatus*, indicating that the *CmHsfs* have remained closely related to those of these five species during the process of evolution. In addition, we found that certain *CmHsf* genes on chromosomes Cm_Chr02, Cm_Chr06, Cm_Chr08, and Cm_Chr016 corresponded to two or more *Hsf* genes in *A. thaliana*. This phenomenon was more fully reflected in the collinear diagram of *C. moschata* with *L. siceraria*, *C. sativus*, *C. maxima* and *C. lanatus*. In general, the collinear relationship between *C. moschata* and *L. siceraria*, *C. sativus*, *C. maxima* or *C. lanatus*) was closer than that for *A. thaliana*, suggesting that these species may have originated from the same ancestor. The collinear analysis showed that *C. moschata* and *L. siceraria*, *C. sativus*, *C. maxima*, and *C. lanatus* had frequent collinearity (Fig. 5), indicating that genes with collinear relationship may have similar functions.

### Expression pattern of *Hsf* genes in *C. moschata*

**Fig. 6:**
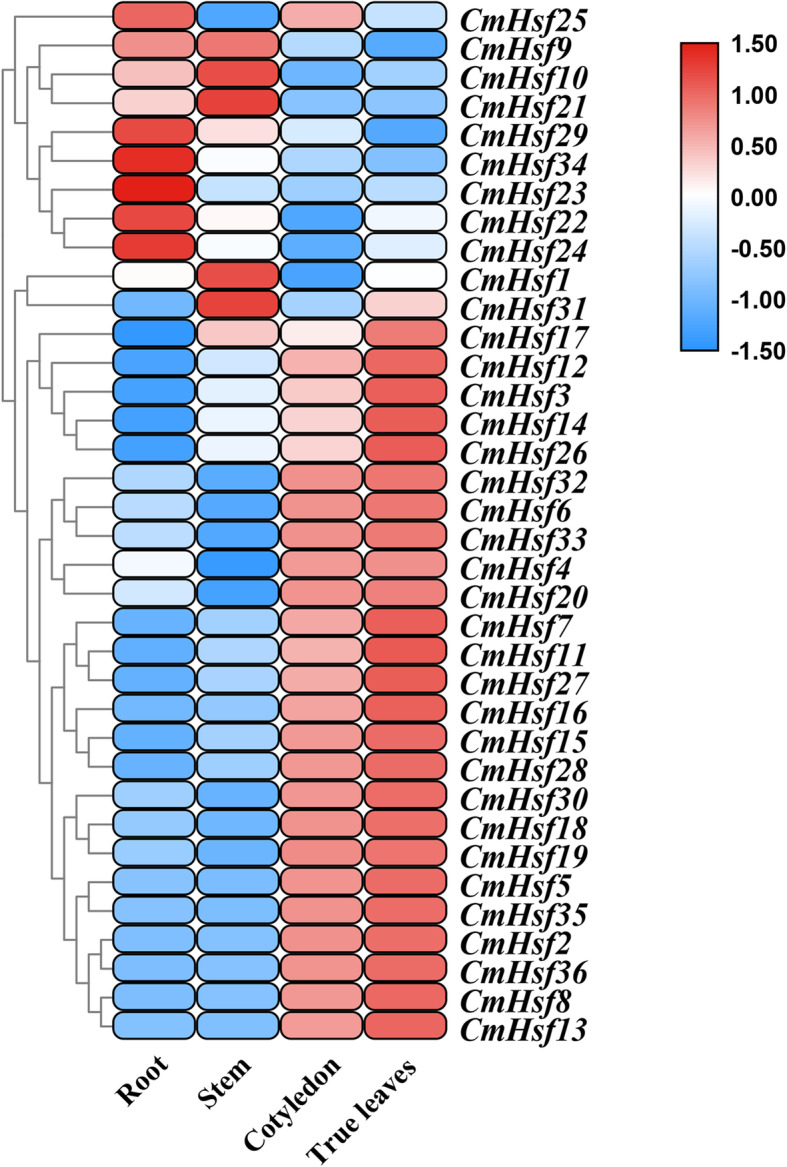
Heat map and hierarchical clustering of 36 *CmHsf* genes in the roots, stems, cotyledons and true leaves. Quantitative real-time PCR was performed in three biological replicates and three technical replicates, and the heat map and hierarchical clustering were constructed by TBtools. The results were calculated via the 2^−ΔΔCt^ method, and the reference gene (*β*-Actin) was used to correct the expression level of target genes. All data were standardized by Log_10_ (2^−ΔΔCt^). The bar on the right of the heat map represented the data that has been converted to Log_10_ (2 ^-ΔΔCt^)

To understand the physiological role of *CmHsfs*, we analysed the expression patterns of 36 heat shock transcription factors in the roots, stems, cotyledons and true leaves of *C. moschata* via quantitative real-time PCR. The transcriptional abundance of 36 *C. moschata* heat shock transcription factors can be obtained from at least one of the four tissues (Fig. 6; Additional file 1: Table S1). Heat map and cluster analyses showed that *21 CmHsfs* were highly expressed in cotyledons and true leaves, such as *CmHsf4*, *CmHsf32*, *CmHsf35*, *CmHsf19* and *CmHsf15*. Two genes (*CmHsf9* and *CmHsf10*) were expressed more highly in the roots and stem than in the cotyledons and true leaves. Some genes were highly expressed only in one tissue. For example, *CmHsf23* was mainly expressed in the roots, and its relative expression level was 100–258 times that in other tissues. Based on the above analysis, 36 heat shock transcription factors showed tissue specificity.

### *Cis*-acting element analysis of *Hsf* genes in *C. moschata*

**Fig. 7:**
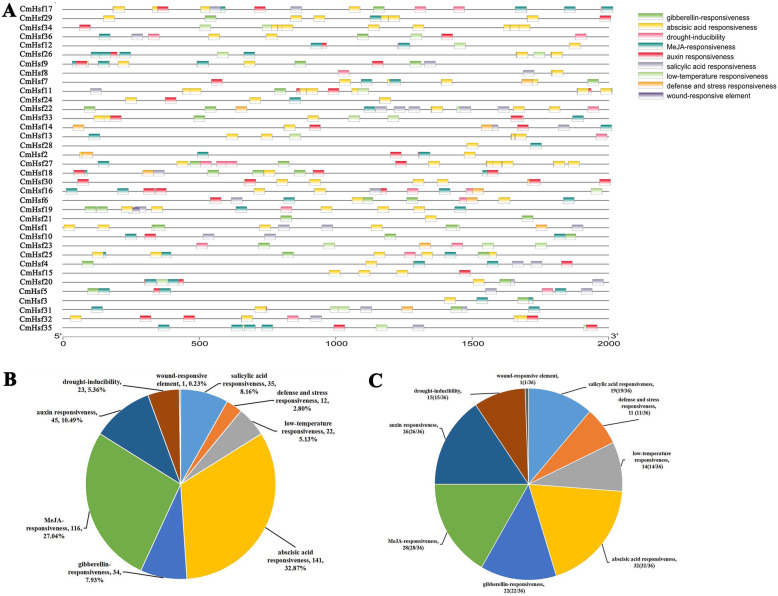
Distribution of *cis*-acting elements in 36 *CmHsfs* and the proportions of corresponding genes in 9 stress response elements. **a.** The *cis*-acting elements of 36 heat shock transcription factors in *C. moschata.* They were predicted by PlantCare program (http://bioinformatics.psb.ugent.be/webtools/plantcare/html/) and visualized by Simple BioSequence Viewer in TBtools. The squares on the right represented *cis*-acting elements that respond to a total of 9 stresses. Different colors indicated *cis*-acting elements that participate in different stresses. The coordinates at the bottom of the figure indicated the length of the gene promoter. The promoter sequence was defined as 2 kb before the start codon. **b.** The distribution of 429 *cis*-acting elements related to 9 abiotic stresses. **c.** The proportion of 36 *CmHsfs* related to 9 abiotic stresses

To explore the potential function of *Hsfs*, the *cis*-elements in the promoters (2 kb before the start codon) of the 36 *Hsf* genes in *C. moschata* were predicted. A total of 429 cis-elements were found among all *CmHsf*s. They were involved in 9 abiotic stresses, including showing salicylic acid responsiveness, defence and stress responsiveness, low-temperature responsiveness, abscisic acid responsiveness, gibberellin responsiveness, MeJA responsiveness, auxin responsiveness, drought inducibility and wound responsiveness (Fig. 7a; Additional file 1: Table S2). A total of 31% of the 429 *cis*-acting elements were involved in abscisic acid responsiveness, which existed in 32 of the 36 *CmHsfs* (Fig. 7b, c). In addition, 27 and 45% of the *cis*-acting elements were MeJA response elements (harboring CGTCA and TGACG motifs) and auxin response elements, respectively (Fig. 7b). Among the 36 heat shock transcription factors, 28 genes were involved in the MeJA response, and 22 genes were involved in the auxin response. A total of 14 heat shock transcription factors exhibited low-temperature response elements. Since the *Hsf* genes involved in abscisic acid responsiveness, low-temperature responsiveness, MeJA responsiveness and auxin responsiveness account for a high proportion of these genes, we speculated that these genes might play important roles in these stresses.

By analyzing the *cis*-acting elements of individual genes, we found that both *CmHsf34* and *CmHsf27* contained 12 abscisic acid response elements (Additional file 1: Table S2). In addition, *CmHsf17*, *CmHsf26*, *CmHsf9* and *CmHsf35* contained 8 MeJA response elements, and *CmHsf23* and *CmHsf35* contained the greatest number (3) of low-temperature response elements, which indicates that these key *CmHsfs* may play an important role in the corresponding stress response.

### The response of *CmHsf* genes to temperature stress

**Fig. 8:**
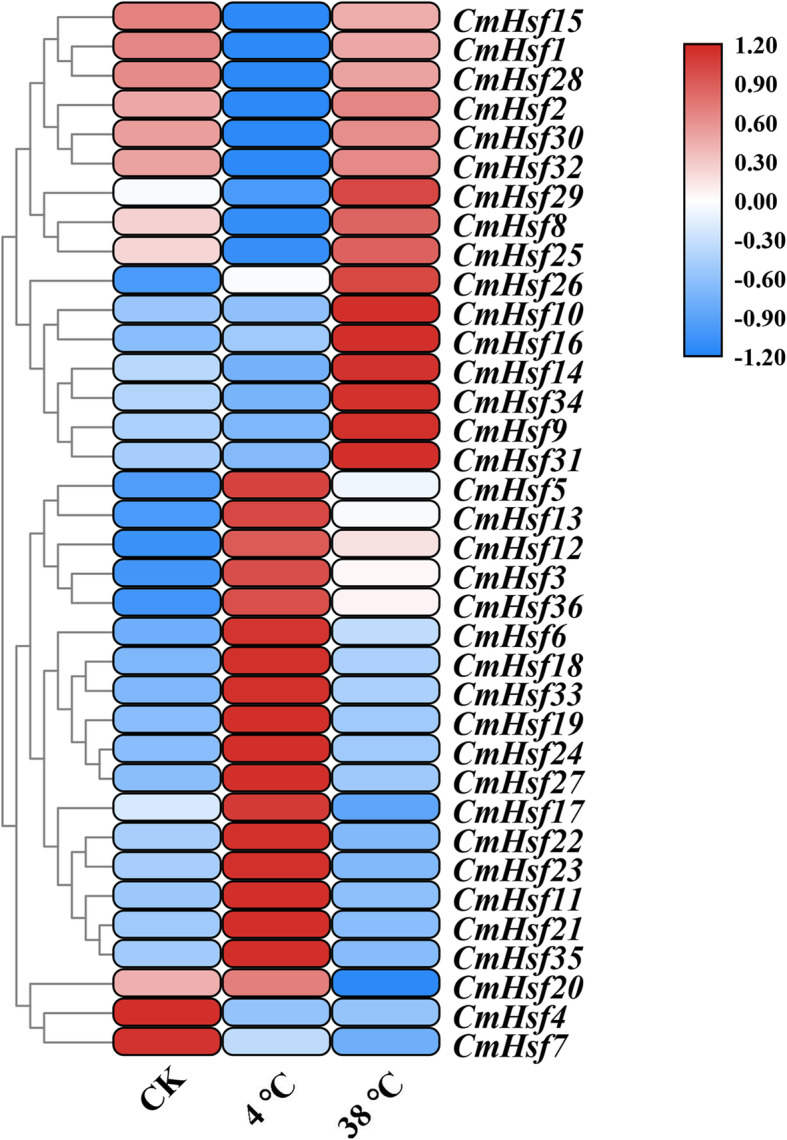
Heat map and hierarchical clustering of 36 *CmHsf* genes in true leaves under cold stress and heat stress. Quantitative real-time PCR and hierarchical clustering were performed in three biological replicates and three technical replicates, and the heat map was constructed by TBtools. The results were calculated via the 2^−ΔΔCt^ method, and the reference gene (*β*-Actin) was used to correct the expression level of target genes. All data were standardized by Log_10_ (2^−ΔΔCt^). The bar on the right of the heat map represented the data that has been converted to Log_10_ (2 ^-ΔΔCt^)

To explore the response of *CmHsfs* to temperature stress, we cultured *C. moschata* seedlings at 4 °C and 38 °C. Under cold treatment, 44% of the *CmHsfs* (16 genes) were significantly upregulated, and 27% of the *CmHsfs* (10 genes) were significantly downregulated (Fig. 8; Additional file 1: Table S3). For instance, *CmHsf3*, *CmHsf5*, *CmHsf23*, *CmHsf24*, *CmHsf27*, *CmHsf35* and *CmHsf36* were highly expressed under cold stress. In addition, the *CmHsf4*, *CmHsf15*, *CmHsf31* and *CmHsf32* genes exhibited low expression levels under cold stress. At the same time, two genes (*CmHsf28* and *CmHsf30*) were not expressed under cold stress, indicating that the expression of these genes may be limited under cold stress. Under heat treatment, 24 genes were significantly upregulated, and 12 genes were significantly downregulated (Fig. 8; Additional file 1: Table S3). The expression levels of *CmHsf9* and *CmHsf31* under heat stress were 128.38 and 66.39 times those in the control plants, respectively, suggesting that these two genes may play important roles under heat stress. Some genes presented low expression levels under heat treatment, such as *CmHsf17*, *CmHsf11*, *CmHsf21*, *CmHsf22*, *CmHsf23* and *CmHsf35*. Considering the expression levels of the *CmHsf* genes under cold and heat stress together, we found that *CmHsf9*, *CmHsf11*, *CmHsf21*, *CmHsf23*, *CmHsf31*, *CmHsf34* and *CmHsf35* showed opposite trends under the two stresses, so we speculate that these genes may play important roles in temperature stress.

### The response of *CmHsf* genes to hormones and salicylic acid

**Fig. 9:**
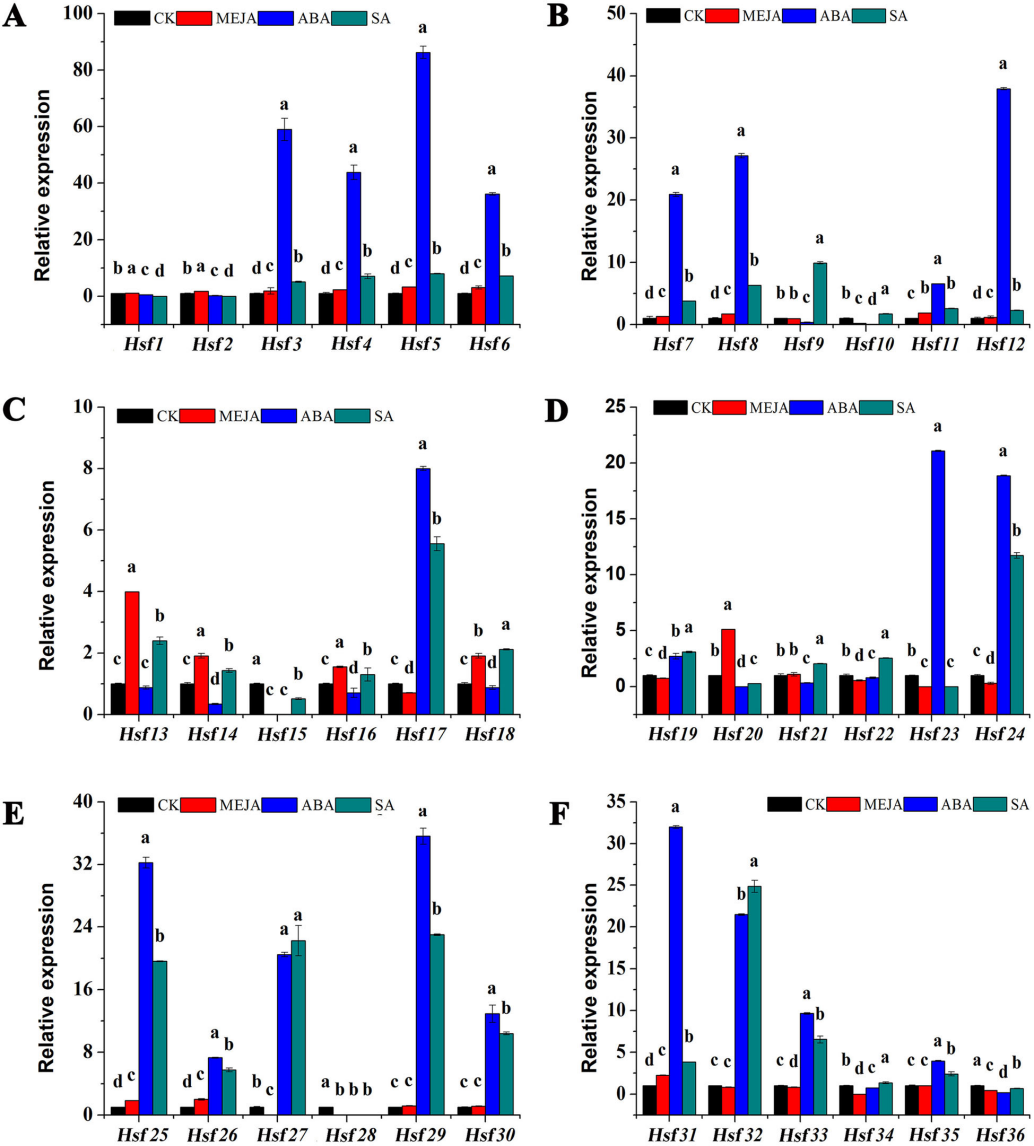
Expression profiles of 36 *CmHsf* genes in true leaves under MeJA, ABA and SA treatments. The data represented the expression levels of *CmHsf* genes at 10 h after the MeJA, ABA and SA treatments. CK referred to untreated plants (control plants) under normal conditions. The results were calculated via the 2^−ΔΔCt^ method, and the reference gene (*β*-Actin) was used to correct the expression level of target genes. The expression level of CK was set as 1. The data were presented as the means of three biological replicates and three technical replicates, and the error bars represented the standard deviations of the means. According to Welch’s t-test, different letters above the bars indicated significant differences (*p* < 0.05) between different treatments

According to the prediction of *cis*-acting elements in the *CmHsf*s promoter, a total of 28, 32, and 19 *CmHsf* genes were found to be involved in the MeJA response, abscisic acid responsiveness and salicylic acid responsiveness, respectively (Fig. 7; Additional file 1: Table S2). Therefore, we analysed the responses of these genes to MeJA, ABA, and SA. The results of qRT-PCR analysis showed that 31 *CmHsf*s responded to MeJA to varying degrees, and the expression of *CmHsf20* was 5.1 times that in the control (Fig. 9; Additional file 1: Table S4). Under ABA treatment, 21 *CmHsf*s were significantly upregulated, and 15 genes were significantly downregulated. The expression levels of *CmHsf3*, *CmHsf4*, *CmHsf5*, *CmHsf6*, *CmHsf7*, *CmHsf8*, *CmHsf12*, *CmHsf25*, *CmHsf29* and *CmHsf31* under ABA stress were 20 ~ 86 times those of the control plants, indicating that these genes play important roles under ABA stress. All *CmHsf*s responded to SA, among which *CmHsf25*, *CmHsf27*, *CmHsf29* and *CmHsf32* were significantly increased under SA treatment, while *CmHsf1*, *CmHsf2*, *CmHsf23* and *CmHsf28* were significantly decreased under SA treatment. Based on the above analysis, we conclude that *CmHsf* family genes are involved in multiple stresses and may play different roles in these stresses.

## Discussion

Heat shock transcription factors are broadly present in all plants and are considered to be important regulators of abiotic stress. The Hsf family has been comprehensively and systematically analyzed in *G. max* [17], *B. rapa* [18], *P. bretschneideri* [19], *S. tuberosum* [20], *V. vinifera* [21] and *B. oleracea* [22]. However, the Hsf family has not been extensively studied in *C. moschata*.

In our study, we identified 36 *Hsf* genes in *C. moschata* via genome-wide analysis (Table 1). The analysis of the physical and chemical properties of the gene family can show the diversity of each member in the process of evolution [45]. Our results showed that the MW and the number of amino acids of 36 CmHsfs vary widely (Table 1), which indicates that *C. moschata* changes in the process of evolution. Most of the CmHsfs exhibited low isoelectric points (*pI*) (average 6.3), this result is similar to the report of Hsf in *C. sativa* [16]. Regardless of the size and domain composition of proteins, the characteristics of low *pI* are preserved, indicating that CmHsf proteins should be negatively charged at physiological pH. Through predictive analysis of subcellular location, it was found that most of the CmHsfs were predicted to be localized to the nucleus (Table 1), which indicated that their functions were indeed as transcription factors. But CmHsf12 and CmHsf17, from the same subfamily, were predicted to be localized to cell membrane, cytoplasm and nucleus, indicate that CmHsf members in the same subfamily do not necessarily correspond to the same subcellular location, and they might have other special function.

The phylogenetic tree divided 36 *CmHsfs* into 3 subfamilies (Subfamily I, Subfamily II and Subfamily III), most of the genes within the same subfamily shared similar gene structures in terms of either exon length or intron number (Fig. 2). Therefore, we speculated that the *CmHsfs* in one branch may have similar functions, and this feature was similar to that previously reported in other species [16, 18]. The structural characteristics of some *CmHsfs* in the same branch are different from those of other *CmHsf* genes, indicating that these genes may have functional diversity. In addition, The *CmHsfs* were also divided into three groups (groups A, B and C) based on the conserved structural characteristics of the DBD and the HR-A/B domain (Table 2). Subfamily II corresponded to group C and subfamily III corresponds to group B, subgroup I contained not only group A genes but also group C genes. Due to the close homology of the genes on the same branch, we speculate that the evolutionary path of the *CmHsfs* has been changing.

The conserved motifs of CmHsfs protein were also predicted and analyzed (Fig. 1). It was found that motifs 1, 2 and 4 exist in all CmHsf proteins (Fig. 1). According to the comprehensive analysis of the motif position and conserved domain position of 36 CmHsf protein, we found that motif 1, motif 2 and motif 3 together formed the DBD, and motif 4 formed the HR-A/B domain (Fig. 1; Table 2). The result is consistent with the previous reports in *Z. mays* [15], *C. sativa* [16], *B. rapa* ssp. *pekinensis* [18], *S. tuberosum* [20], which indicates that *CmHsfs* may have similar functional characteristics.

In some species, the number of members of a specific gene family is considered to be the result of natural evolution. At the same time, the diversity of gene family members is generally due to genome recombination and amplification [46]. Chromosomal segmental duplications and individual gene duplications are a major driving force in the genome evolution process [47]. Compared with the 25 reported *ZmHsf*s [15], 21 *CsHsf*s [16] and 31 *PtHsf*s [14], we found that the number of *Hsf* genes in *C. moschata* is greater than those in *Z. mays, C. sativa* and *P. trichocarpa*. Genome sizes vary significantly in these species; for instance, the genome size of *C. moschata* is 197.83 Mb, and that of *Z. mays* is 2300 Mb. The maize genome size is 11 times that of *C. moschata.* However, the number of maize *Hsf* genes is much lower than the number of *Hsf* genes in *C. moschata*. The reason for this difference might be that although two rounds of gene duplication occurred in the *Z. mays* genome during its evolution [48, 49], the *Hsf* genes of *Z. mays* underwent large gene losses. In addition, the genome of *C. moschata* also underwent a whole-genome duplication (WGD) event during the phylogeny of the species [43]. For *C. sativa*, the genome size is 350 Mb, but 21 *CsHsfs* was less than the number of *CmHsfs*. We speculated that gene duplication promotes the amplification of *CmHsf* genes [43] or gene degeneration and mutation promotes the reduction of the number of *CsHsf* genes [16], ultimately resulting in the number of *CmHsf* genes more than that of other plants.

In this study, all *CmHsf* gene pairs were found to have experienced segmental duplication events, with no tandem duplication events, indicating that segmental duplication has played an important role in the evolution of the *C. moschata Hsf* gene family (Fig. 3). The *Ka* and *Ks* ratios of all duplicated pairs indicated that these gene pairs were under purifying selection. Additionally, the relatively high Ka/Ks ratios for *CmHsf12*-*CmHsf10* suggested that they have experienced rapid evolution (Table 3).

A study proposes three hypotheses to explain the fate of duplicated genes: (1) In the process of plant evolution, sometimes gene degeneration and mutation occur, which often leads to the loss of copy function of some duplicated genes. (2) Due to the diversity and directionality of mutations, one copy of the duplicated gene may mutate and retain its new function during evolution, while the other copy retains its original function. This process is called new functionalization. (3) Two copies of the duplicated gene may mutate to obtain different functions, which is called subfunctionalization [50]. According to the different expression patterns of *CmHsf26* and *CmHsf9* genes, it can be inferred that there are differences between the duplicated genes. *CmHsf26* is highly expressed in cotyledon and true leaf, while *CmHsf9* gene is highly expressed in root and stem (Fig. 6). Their gene structure and motif composition are similar, which indicates that the subfunctionalization of duplicated genes in *CmHsf* gene family may change the gene expression pattern (Fig. 1; Fig. 2). In addition, the duplicated genes *CmHsf30* and *CmHsf16* have similar intron-exon structure, the same motif component, and the similar tissue expression pattern, but there are obvious differences in temperature stress and hormone treatment (Fig. 2; Fig. 6; Fig. 8; Fig. 9), which indicates that the new functionalization of the duplicated genes in the *CmHsf* gene family may play a key role. The collinear analysis showed that *C. moschata* had frequent collinearity with *L. siceraria*, *C. sativus*, *C. maxima*, and *C. lanatus* (Fig. 5), indicating that genes with collinear relationship may have similar functions.

*Cis*-acting elements are essential for gene expression, and their numbers are correlated with gene expression intensity [51, 52]. *CmHsf23* and *CmHsf35* contain three low-temperature response elements (Fig. 7), which mean that *CmHsf23* and *CmHsf35* may play key roles under low-temperature stress. The qRT-PCR results showed that *CmHsf23*, *CmHsf21*, *CmHsf11*, and *CmHsf35* were significantly upregulated under low temperature, and the expression profiles of these genes showed opposite trends under high-temperature stress, which further verified the response of these genes to temperature stress (Fig. 8). However, *CmHsf13*, *CmHsf36*, *CmHsf3* and *CmHsf5* were significantly induced under cold stress and heat stress (Fig. 8), and their responses were more prominent under cold stress, which indicated that these genes were highly sensitive to temperature and might play a key role under temperature stress. The prediction of *cis*-acting elements showed that the promoters of 28 *CmHsf* genes contained MeJA response elements (Fig. 7), and qRT-PCR analysis showed that the expression levels of 31 genes changed to varying degrees under MeJA treatment (Fig. 9). However, from the relative expression values, we found that the *CmHsfs* responded less to MeJA than to ABA and SA (Fig. 9). Therefore, we concluded that *C. moschata* Hsf family genes were mainly involved in the respond to ABA and SA.

## Conclusions

In summary, we identified 36 *Hsfs* in the *Cucurbita moschata* genome based on a thorough analysis and provided genetic information such as chromosome locations and exon-intron structures, conserved domains, and duplicated genes. We specifically examined the expression profiles of these *CmHsfs* in different tissues. At the same time, we examined the responses of *CmHsfs* to multiple stresses, and several key genes were found to respond to adverse environments.

## Methods

### Sequence retrieval from the *Cucurbit* genomics database and physicochemical characterization

To identify the heat shock transcription factor family in *C. moschata*, the genome was downloaded from the *Cucurbit* genomics database (CuGenDB, http://cucurbitgenomics.org/) [43]. A total of 25 *A. thaliana Hsf* genes were obtained from the NCBI database by using their gene IDs from *A. thaliana* references [26]. We used 25 AtHsf proteins as queries to search against the *Cucurbit* genomics database using BLASTP with an *e*-value cut-off of 1 × e^− 10^. To eliminate false positives, sequences were discarded if they constituted < 70% of the corresponding *A. thaliana* Hsf protein. SMART (http://smart.embl-heidelberg.de/) [53] and MARCOIL (http://toolkit.tuebingen.mpg.de/marcoil) [19] were used to predict the DBDs and HR-A/B domains. After the removal of the same genes, the remaining genes were identified as *CmHsf* genes. The coding sequence and protein sequence information for each of the CmHsfs were shown in Additional file 1: Table S6.

The physical and chemical characteristics of the heat shock transcription factors, including their theoretical molecular weight (MW), theoretical isoelectric point (*pI*) and the number of amino acids, were analyzed with ExPASy (http://web.expasy.org/tools/) [54]. Information on *CmHsf* genes including their chromosomal distribution, their start and the end positions on the chromosomes were extracted from the *Cucurbit* genomics database, and their subcellular locations were predicted with Plant-mPLoc [55].

### Phylogenetic tree construction

To reveal the phylogenetic relationships of *Hsf* genes in *C. moschata*, an unrooted phylogenetic tree was constructed with MEGA 5.0 [56] according to the similarity of full-length amino acid sequence of 36 CmHsfs. In addition, the phylogenetic relationship of Hsf protein from *C. moschata*, *C. sativa* and *A. thaliana* was also constructed by MEGA 5.0. The protein sequences of 21 CsHsfs and 22 AtHsfs were obtained based on previous literature [44, 57]. The unrooted Neighbor-Joining (NJ) method was used to construct the phylogenetic tree, and the bootstrap values were obtained using 1000 replicates with the pairwise deletion option.

### Analysis of conserved domains and gene structure

The conserved motifs of Hsf in *C. moschata* were obtained on the Multiple Expectation Maximization or Motif Elicitation (MEME, http://meme-suite.org/) [58] using the protein sequences, and the LOGOs (Additional file 2: Fig. S2) of the protein motifs were also obtained with MEME. The NLSs and NESs of the heat shock transcription factors were predicted by using cNLS Mapper [59] and the NetNES 1.1 Server [60], respectively. The exon-intron structures were obtained from GSDS (Gene Structure Display Server, http: //gsds.cbi. pku.edu.cn/) [61] by comparing the cDNA sequences and its corresponding genomic DNA sequences of CmHsfs members.

### Gene duplication and gene collinearity analysis

The chromosomal locations of the *CmHsf* genes were mapped and imaged with visualization tools (http://visualization.ritchielab.psu.edu/home/index) based on their initial positional information obtained from *C. moschata* (CuGenDB, http://cucurbitgenomics.org/). To identify gene duplications, all CDS sequences of *C. moschata Hsf* genes were subjected to BLAST searches against each other (Identity > 85%, E-value <1e^− 10^) by using the Local Blast program. Gene alignment coverage was then acquired by pair-wise alignment using the previously calculated method: Gene alignment coverage = (alignment length - mismatches)/length of the longer gene. Pairs were considered duplications when the gene alignment coverage was greater than 0.75. Moreover, two genes that were separated by several genes in a 100-kb were named as tandemly duplicated genes [62]. To estimate the divergence of these duplicated *CmHsf* genes, we used the KaKs calculator to calculate the synonymous substitution ratio (*Ks*) according to the method of Gojo-bori and Nei [63]. To avoid the saturation of substitutions, we required that Ks values > 2.0 must be discarded [64, 65]. The divergence time (T) was computed according to the formula (T = Ks/2λ × 10^− 6^ million years ago (Mya), λ = 1.5 × 10^− 8^) in the previous literature [66]. The criteria for identifying gene collinearity were based on previous reports [67], and the synteny relationships between the heat shock transcription factors of *C. moschata* and those of other species (*A. thaliana*, *C. sativus*, *C. maxima*, *C. lanatus*, *L. siceraria*) were constructed using Advanced Circos program in TBtools [68]*.*

### Analysis of *cis*-acting elements of *CmHsf* gene promoters

The promoter sequences (2 kb before the start codon) of all *CmHsf* genes were extracted from the *Cucurbit* genome database (http://cucurbitgenomics.org/), and we predicted the promoter *cis*-acting elements of *CmHsfs* by using PlantCare program (http://bioinformatics.psb.ugent.be/webtools/plantcare/html/) [69] and visualized by Simple BioSequence Viewer in TBtools [68].

### Plant material, growth conditions and stress treatment

The *C. moschata* variety “Tianmi 1” was used as the study material. The seeds were provided by the pumpkin team of School of Horticulture and Landscape Architecture, Henan Institute of Science and Technology. The seeds were sown in a tray containing a vermiculite-matrix (2:1) mixture and grown in a plant growth chamber. The artificial growth conditions were set as light intensity of 350 μmol/m^2^/sec, 25 °C 16 h light / 16 °C 8 h dark and 65% relative humidity. We sampled and analyzed different tissues (roots, stems, cotyledons and true leaves) of two-month-old seedlings. In addition, some of the seedlings were transferred to 38 °C for 6 h heat treatment, or transferred to 4 °C for 6 h cold treatment. Another portion of the seedlings was cultured in 1/2 Hoagland solution, pH 6.5. After 5 days of adaptation, the plants were cultured with the following treatments: (1) control (untreated plants); (2) 1 mM MeJA; (3) 5 mM salicylic acid (SA); (4) 100 μM abscisic acid (ABA) [70]. Leaf samples were collected at 10 h after the above treatments. Control and stress-treated samples were frozen in liquid nitrogen and stored at − 70 °C for further analysis.

### RNA extraction, reverse transcription and qRT-PCR analysis

Total RNA was extracted from the frozen samples according to the instructions of the RNA kit (Tiangen, Beijing). Moreover, the RNA was isolated and then reverse transcribed into cDNA using a Prime Script RT reagent kit (TaKaRa, Dalian, China). Finally, quantitative real-time PCR was performed using the SYBR Premix ExTaq kit (TaKaRa, Dalian). To verify the specificity of gene primers, the target genes and the reference gene (*β*-Actin) primers (Additional file 1: Table S5) were aligned at the *Cucurbit* genome database. The qRT-PCR analysis was performed on an ABI7500 Real-Time PCR System (Applied Biosystems) with the following cycling profile: stage 1, 95 °C 20 s; stage 2, 95 °C 3 s, 60 °C 30 s (40 cycles); stage 3, 95 °C 15 s, 60 °C 1 min, 95 °C 15 s. Stage 3 was used to perform a melting curve. Experimental repeats were run for three technical and three biological replicates. The relative gene expression was calculated according to the 2^-ΔΔCt^ method.

## Supplementary information

**Additional file 1: Table S1.** The data of expression levels of 36 *CmHsf* genes in root, stem, cotyledon and true leaves. The results were calculated via the 2^−ΔΔCt^ method. **Table S2.** Statistics for the number of *cis*-acting elements in the 36 *CmHsf* gene promoters. **Table S3.** The data of expression levels of 36 *CmHsf* genes in leaves under temperature stress. CK referred to the untreated plants at 25 °C. The data represented the expression levels of *CmHsf* genes at 6 h after cold stress (4 °C) and heat stress (38 °C). The results were calculated via the 2^−ΔΔCt^ method. **Table S4.** The data of expression profiles of 36 *CmHsf* genes in true leaves under MeJA, ABA and SA treatments. CK referred to untreated plants in this figure. The data represented the expression levels of *CmHsf* genes at 10 h after the MeJA, ABA and SA treatments. The results were calculated via the 2^−ΔΔCt^ method. **Table S5.** List of primer sequences used for the tissue-specific analysis of 36 *CmHsf* genes. **Table S6.** The coding sequence and protein sequence information for each of the CmHsfs.

**Additional file 2: Figure. S1.** Multiple sequence alignment analysis and the secondary structure elements of DBD in CmHsf proteins. Sequence alignments were performed using Clustal X 2.0. Different background colors indicated different amino acids. “*” meant that the amino acid sequences of different Hsf proteins are highly consistent. The secondary structure elements of DBD (*α*1-*β*1-*β*2-*α*2-*α*3-*β*3-*β*4) were shown above the alignment. The secondary structure was predicted by SOPMA secondary structure prediction software. Cylindrical tubes represented *a*-helices or *β*-sheets. **Figure. S2.** Detailed information about the 10 motifs identified in CmHsf proteins. The LOGOs of the protein motifs were also obtained with Multiple Expectation Maximization or Motif Elicitation (MEME, http://meme-suite.org/).

## Data Availability

The *Cucurbita moschata Hsf* gene and protein sequences were downloaded from the *Cucurbit* genomics database (CuGenDB, http://cucurbitgenomics.org/). All of the data and materials supporting our research findings are contained in the methods section of the manuscript. Details are provided in the attached additional files.

## Abbreviations

*Hsfs*: Heat shock transcription factors
DBD: DNA-binding domain
OD: Oligomerization domain
NLS: Nuclear localization signal
NES: Nuclear export signal
HSE: Heat shock element
RD: Repressor domain
Hsp: Heat shock protein
*Cm*: *C. moschata*
kDa: KiloDaltons
*pI*: Isoelectric points
WGD: Whole-genome duplication
Mw: Molecular weight
MEME: Multiple expectation maximization or motif elicitation
CDS: Coding domain sequence
*Ks*: Synonymous substitution ratio
Mya: Million years ago
SA: Salicylic acid
ABA: Abscisic acid
