# Supplementary figures and images for: Genome-wide characterization and expression analysis of the heat shock transcription factor family in pumpkin (Cucurbita moschata)
Source: BMC Plant Biol. 2020 Oct 14;20:471. doi: 10.1186/s12870-020-02683-y (PMC7557022; doi:10.1186/s12870-020-02683-y)

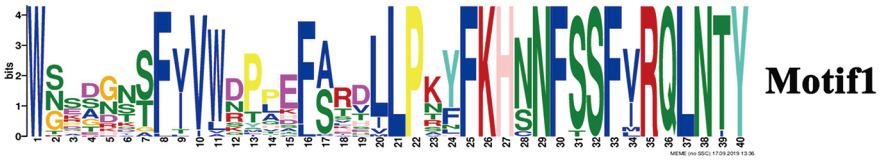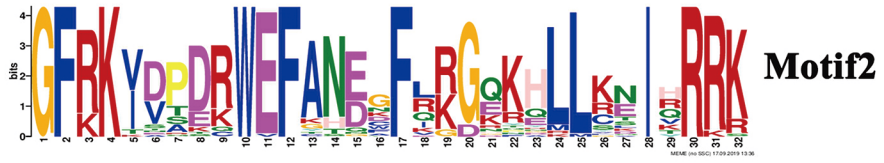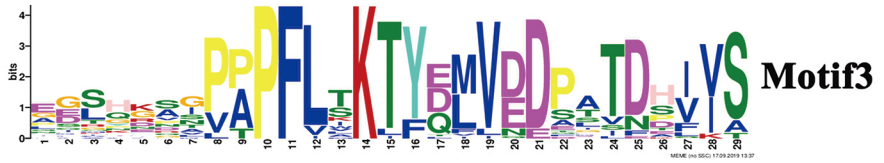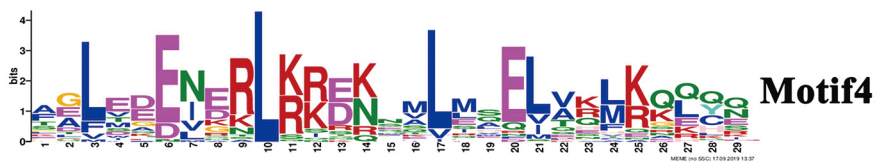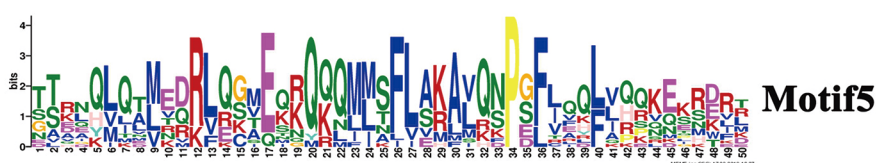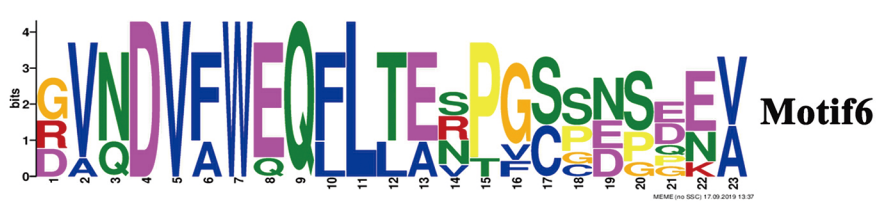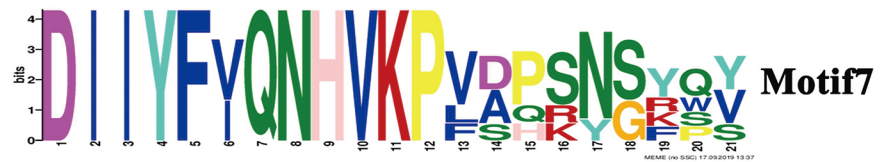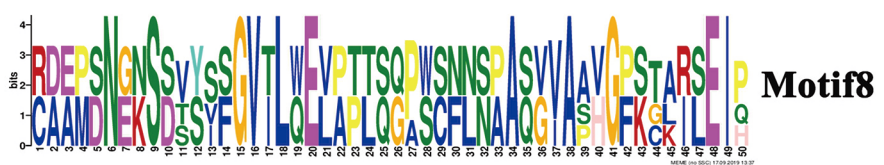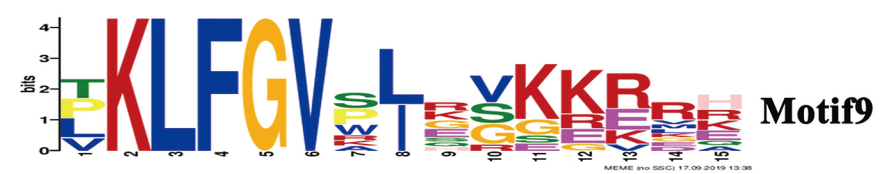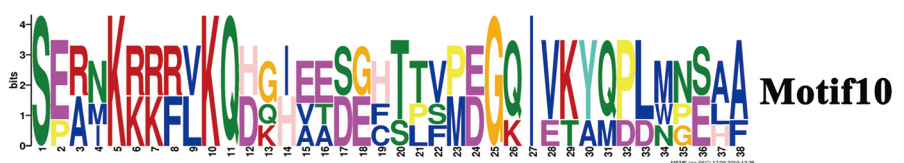

Supplement: Supplementary file 2 — Additional file 2: Figure. S1. Multiple sequence alignment analysis and the secondary structure elements of DBD in CmHsf proteins. Sequence alignments were performed using Clustal X 2.0. Different background colors indicated different amino acids. “*” meant that the amino acid sequences of different Hsf proteins are highly consistent. The secondary structure elements of DBD (α1-β1-β2-α2-α3-β3-β4) were shown above the alignment. The secondary structure was predicted by SOPMA secondary structure prediction software. Cylindrical tubes represented a-helices or β-sheets. Figure. S2. Detailed information about the 10 motifs identified in CmHsf proteins. The LOGOs of the protein motifs were also obtained with Multiple Expectation Maximization or Motif Elicitation (MEME, http://meme-suite.org/). [file 12870_2020_2683_MOESM2_ESM.zip › Fig. S2.pdf]
